# Supplementary material for: Are carbon emissions trading and green financial instruments synergistic? -Comprehensive quantitative research based on content analysis
Source: PLoS One. 2024 Mar 7;19(3):e0298601. doi: 10.1371/journal.pone.0298601 (PMC10919601; doi:10.1371/journal.pone.0298601)
Supplement: S1 Data — (ZIP) [file pone.0298601.s001.zip › Data/地级市Z_F工作报告.zip!/╕╩╦α/╟∞╤⌠╩╨/└┤╫╘╣1⁄2╓┌║┼í╛┬φ┐╦╩2╛▌═°í┐_2015.docx]

各位代表：

　　现在，我代表市人民政府向大会报告工作，请予审议，并请各位政协委员和列席人员提出意见。

　　2014年工作的回顾

　　2014年，在省委、省政府和市委的坚强领导下，我们深入贯彻党的十八大和十八届三中、四中全会精神，牢牢把握稳中求进、改革创新的总基调、总要求，攻坚克难，开拓进取，全市经济平稳增长，民生持续改善，社会和谐稳定。

　　经济发展进入运行稳健、结构优化的新常态。积极应对经济下行压力加大、能源市场波动等不利影响，统筹推进稳增长、调结构、转方式、惠民生、防风险的一系列措施，经济增长处在合理区间,发展的质量和效益不断提升，完成了市人大常委会批准调整后的各项预期目标。预计全年生产总值完成669亿元，增长10.5%。规模以上工业增加值369亿元，增长11.3%。地方固定资产投资总额981亿元，增长23%。社会消费品零售总额165亿元，增长13%。财政大口径收入149.25亿元，公共预算收入61.54亿元,同口径比较分别增长1.1%和8.27%。城镇居民人均可支配收入21106元，增长12.5%。农民人均纯收入5612元，增长14.8%。

　　富民产业步入规模扩大、提质增效的新阶段。粮食生产喜获丰收，总产量164.2万吨，创历史新高。畜牧产业发展取得突破，“中盛模式”带动肉鸡产业跨越发展，引领我市现代畜牧业发展迈上新台阶。肉羊养殖规模快速扩大，成为群众脱贫致富的短、平、快项目。新增各类规模养殖场235个。苹果产业效益明显提升，“庆阳苹果”品牌效应初步显现，预计销售收入23.7亿元。苗林产业走出了大地增绿、农民增收的“双赢”之路，政策创新激发了广大群众参与开发的强大活力，全年造林113万亩。蔬菜产业规模不断扩张，设施蔬菜种植面积达到13万亩。产业化经营水平持续提升，新建、改扩建农产品加工企业30户，新增农民专业合作社1122个。扶贫攻坚成效明显，投入各类资金72.8亿元，实施整村推进120处、易地搬迁103处，减少贫困人口13.54万人。第三产业持续发展，交通运输、商贸物流等服务业平稳增长，汽车、信息、旅游消费日趋活跃。陇禧、宁南物流园基本建成，市区10个专业市场开工建设。南梁红色旅游景区建成投用，庆阳民俗文化产业园、太白陕甘红军纪念园、山城堡纪念园等景区建设进展良好。

　　优势资源取得链式开发、转型升级的新进展。陇东能源基地开发规划和“两个千亿级产业链”建设方案分别获得国家能源局和省政府批复。油气开发再上台阶，原油产量达到722.9万吨、加工量330.4万吨，庆阳石化600万吨炼油升级改造和天然气综合利用项目稳步推进。煤炭开发步伐加快，3个区块完成普查、详查；刘园子煤矿建成投产，产煤50万吨；核桃峪煤矿“四井”贯通，甜水堡2号矿井即将建成；新庄、马福川2个矿井和正宁电厂获得国家发改委核准，环县电厂已列入省内火电建设规划。风电基地建设快速推进，毛井一期40万千瓦项目即将建成，毛井二期40万千瓦和甜水堡、紫坊、乔河3个5万千瓦项目取得“路条”。“一区四园”建设进展顺利，庆阳经济技术开发区发展规划已经省政府批准并上报国务院待批，四个园区“七通一平”基础建设加快推进，实施各类项目49个，新增入驻企业10户。

　　基础建设实现投入加大、进度加快的新突破。“3341”项目工程深入实施，全年建设500万元以上项目2459个，其中亿元以上项目102个，是项目实施规模、建设体量最大的一年。交通建设完成投资23.2亿元，机场航站楼主体工程完工，开通了庆阳至上海、银川新航线。银西铁路获得国家批复，甜罗高速公路可研上报国家发改委、交通运输部待批。新修通村油路（水泥路）1669公里，是我市历史上投入最大、建设里程最长的一年。水利建设完成投资9.4亿元，西峰雨洪集蓄保塬生态项目主体工程基本建成，莲花寺、小盘河水库和巴家嘴水库新增调蓄工程可研报告得到批复。马莲河枢纽工程进入国务院重点支持的全国重大水利工程名录，前期工作有序推进。建设农村饮水工程2万处，解决了18.8万人饮水问题。城乡电网建设完成投资4.7亿元，环县330千伏输变电工程完成年度建设计划，3个110千伏输变电工程建成投运，新增供电能力17.4万千伏安。改造低电压村（组）896个、农电户4.5万户。生态环境建设完成投资13亿元，“再造一个子午岭”和“固沟保塬”工程扎实推进。治理流域380平方公里，新修梯田31.5万亩。创建国家和省级生态乡镇80个，建设美丽示范村和环境整洁村136个。城镇建设完成投资27亿元，实施道路、供排水、供热、污水和垃圾处理等基础设施项目160个。市县29个片区、112万平方米棚户区改造全面拉开。建设保障性住房1.46万套，改造农村危房（窑）1.53万户。13个城市综合体项目启动实施，县城旧城改造、新区开发和重点小城镇建设步伐加快，城镇化率提高2个百分点。

　　改革开放开启体制创新、活力增强的新局面。行政审批制度改革力度加大，承接省级下放行政审批项目80项，市级取消调整42项，累计减幅达到78%。进驻政务大厅的服务事项344项。工商注册便利化改革成效明显，新增各类市场主体1.6万户。市、县（区）政府机构改革进展顺利，撤销整合市直部门（单位）8个。95%的行政村建立了便民服务中心，打通了服务群众的“最后一公里”。财税管理制度改革加快推进，国库集中支付率达到94.6%，消化逾期历史债务10%。混合所有制经济发展取得新突破，庆阳能源化工集团吸纳多种经济成分参股投资，成立股份制子公司11个，吸引社会资本5.8亿元，承接央企辅业、服务资源开发的能力快速提升。公交体制改革取得成效，公共交通集团组建运营，新增城区公交线路5条，投放节能环保公交车68辆、出租车200辆，为市民出行创造了便利条件。金融服务体系进一步完善，交通银行庆阳分行开业运营，甘肃银行庆阳分行实现县域机构全覆盖。融资渠道不断拓宽，直接融资规模达到27亿多元。农村综合改革稳步推进，累计流转耕地108万亩、林地33.1万亩，国有林场改革进展顺利。耕地保护利用不断加强，陇东百万亩土地整治庆阳片区项目顺利实施。开放合作迈出新步伐，与意大利卡布拉罗拉市签署了6个经济合作项目，在保加利亚、斯洛伐克、奥地利、新加坡等国家开展了经贸洽谈活动。全市完成外贸出口7000万美元。招商引资成效明显，积极参加重点节会招商，开展“千名陇商回家乡”活动，实施招商引资项目301个，到位资金591.6亿元,增长31%。

　　社会事业呈现服务加强、全面进步的新气象。教育质量稳步提升，高考录取率83.19%，高出全省6.09个百分点；改造农村薄弱学校258所，建设农村学校小伙房757个，新建改扩建乡镇幼儿园37所。庆阳职业技术学院获省政府批复。科技推广成效明显，实施农业科技创新项目52项，完成科技开发项目153项，科技贡献率达到50%。医疗卫生服务水平不断提高，实施卫生基础项目332个，公立医院改革列为全国试点，县级综合医院、乡镇卫生院住院费用和药品费用明显下降。计划生育工作完成了年度人口控制目标。

　　文化事业繁荣发展，电视专题片《黄土大塬》在央视播出受到好评，编排历史陇剧9部，拍摄电影6部，新建乡村舞台507个、文化集市48个，群众文化活动丰富多彩。承办了全省第8届少数民族运动会，在第十三届全省运动会上取得了优异成绩。民生实事办理扎实有效，省、市确定的38件民生实事落实到位，“五个百”便民工程加快实施。城乡社会保障统筹推进，城乡低保、农村五保供养和社会保险待遇进一步提高。创业促就业和高校毕业生安置富有成效，新增公益性岗位1168个，城镇新增就业5.6万人，城镇登记失业率控制在2.27%。完成城乡居民技能培训14.6万人，劳务输转60.7万人。社会治理持续加强，安全生产大检查、打非治违、隐患排查治理、农民工工资清欠四个专项行动深入开展，矛盾纠纷排查调处和信访工作扎实有效，社会大局和谐稳定。质量监督、国防动员、双拥、统计、规划、地震、档案、老龄、残疾人等工作取得了新进步。

　　政风建设取得效能提升、环境优化的新成效。政府系统党的群众路线教育实践活动深入开展，“四风”问题得到有效遏制。效能风暴行动深入推进，政府效能明显提高，法治政府建设稳步推进。办理人大代表建议105件、政协委员提案146件。全面贯彻中央“八项规定”、省委“双十条”规定和市委“实施细则”，廉政建设责任和廉政风险防控措施有效落实，审计、监察和政务公开力度加大，政府工作人员艰苦奋斗、勤俭节约的意识增强。全市“三公”经费同比下降38.8%，会议费下降46.2%。

　　各位代表，过去一年的工作能够在发展中突破、在挑战中前行，我们深切地体会到：一是牢牢把握稳中求进总基调，积极适应发展新常态，稳增长、调结构、促发展，开启了推进转型升级的新征程；二是坚持改革创新的办法破除体制机制障碍，抓产业、惠民生、促和谐，创造了促进科学发展的新业绩；三是充分利用一切有利机遇，抓谋划、建项目、争资金，一些事关庆阳长远发展的重大项目取得突破性进展，强化了富民兴市的新支撑；四是弘扬南梁精神，践行群众路线，兴办民生实事，集聚了干部群众支持发展、参与发展的新活力；五是大力倡导实干兴市之风，抓落实，干到位，求实效，营造了风清气正、干事创业的新局面。

　　各位代表，我市经济社会发展取得的成绩来之不易，这是省委、省政府和市委正确领导的结果，是市人大、市政协监督支持的结果，是全市广大干部群众共同努力的结果。在此，我谨代表市人民政府，向各位代表和政协委员，向各民主党派、工商联、人民团体和无党派人士，向离退休老同志，向驻庆人民解放军、武警官兵和中央、省属单位，向奋战在全市各条战线上的劳动者，向所有关心、支持庆阳发展的社会各界人士，致以崇高的敬意和衷心的感谢！

　　同时，我们清醒认识到，前进道路上还有不少困难和问题。从目标任务看，一些重大项目、重点工程未能实现预期，原油生产和加工量未完成年初计划。从体制机制看，农村“三权”改革、资源市场改革等一些改革任务推进缓慢，融资难题和结构性矛盾还没有得到有效解决。从民生保障看，公共服务水平较低，食品安全、医疗服务、环境保护、城市管理等方面与群众的期盼还有差距。从干部作风看，一些政府工作人员担当意识不强，工作效率不高、落实不力，难以适应新形势下经济社会发展的需要。对存在的问题，政府要先从自身找原因、想办法，牢记责任使命，扎实有效解决，决不辜负人民的厚望。

　　2015年主要工作任务

　　2015年，是全面深化改革的关键之年，是全面推进依法治市的开局之年，是完成“十二五”规划的收官之年，是谋划“十三五”发展的重要之年。根据市委“紧盯一个目标、强化一个动力、创建三个生态、实施四大战略、建设十项工程”的发展思路，今年政府工作的总体要求是：全面贯彻落实党的十八大、十八届三中、四中全会和中央、全省经济工作会议精神，坚持稳中求进工作总基调，以全面深化改革为动力，以提高经济发展质量和效益为中心，打好交通大会战，突出扶贫攻坚，突出项目建设，着力稳增长、调结构、转方式，着力打基础、破瓶颈、谋长远，着力察民情、解民忧、惠民生，加快建设幸福美好新庆阳。

　　经济社会发展主要预期目标是：生产总值增长9%，固定资产投资总额增长18%，规模以上工业增加值增长10%，社会消费品零售总额增长12%，财政大口径收入和公共预算收入均增长10%，城镇居民人均可支配收入增长11%，农民人均纯收入增长13%，人口自然增长率控制在7.8‰以内，居民消费价格总水平涨幅控制在3%以内，单位GDP能耗、主要污染物排放完成“十二五”控制指标。

　　上述预期目标，体现了新常态的趋势性、稳增长的必要性、调结构的紧迫性。我们将正视和积极应对新常态下面临的新情况、新变化，全力抓好“十二五”规划的任务落实，认真编制“十三五”发展规划，精心谋划、统筹推进重点工程、重大项目和重要工作，全面完成今年的预期目标，为努力同全国、全省一道全面建成小康社会奠定坚实基础。

　　重点抓好七个方面的工作：

　　（一）以扶贫攻坚为总揽，持续抓好“三农”工作。把扶贫攻坚作为最大的政治和最紧迫的任务，全民动员、精准施策，公共资源向农村配置，项目向农村整合，资金向农村倾斜，啃下“硬骨头”，打好“大会战”。

　　着力改善农村基础条件。新修农村公路2700公里，实现80%的行政村通油路（水泥路）。新建和改造一批农村小型水利工程，确保5万农村人口实现安全饮水。新建改造10千伏线路852公里，治理低电压村组236个，完成447个村组通动力电。按照农民自愿、先易后难、突出重点的原则，整合资源、逐村推进，实施移民搬迁5300户、2.4万人。

　　培育壮大特色富民产业。坚持以“五变”促增收，转变农业发展方式，促进农业结构向优质特色产品调，向种、养、加、销全产业链调。草畜产业，提升肉鸡“三链”生产经营水平，力争年产能达到5000万只、出栏3600万只。争取新上200万只肉羊屠宰分割项目，新建规模养殖场和养殖小区200个，种植（更新）紫花苜蓿100万亩。苹果产业，坚持抓栽植扩规模，抓管理提品质，抓宣传促销售，新栽苹果10万亩，打响“庆阳苹果”品牌。苗林产业，按照“因地制宜、适地适树”的原则，推动种苗培育和荒山造林同步协调发展，全年造林100万亩以上。蔬菜产业，加快推进高原夏菜和设施蔬菜基地建设，蔬菜面积稳定在100万亩以上，其中设施种植达到14万亩。大力推广“中盛模式”，扶强做大年销售收入过亿元的农字号企业10户以上，新增各类农民专业合作组织200个，让“中盛模式”在各个县区、各个产业落地生根、开花结果。大力发展农产品精深加工业,积极拓展优势资源开发配套服务业，引导农民向二三产业转移、向中心城镇集中，减少农民、富裕农民。强化粮食安全保障，落实全膜双垄沟播面积350万亩以上，确保粮食总产量稳定在150万吨以上。

　　持续提高农民致富技能。整合扶贫、农牧、职教、科技、人社等资源，创新培训方式，强化技能培训，提升农民科技、文明、文化和法律素质。全年培训农村劳动力10万人，其中培训农村实用人才、种养大户、农民合作社负责人1000人。发挥“两后生”和致富领军人才的示范引导作用，鼓励支持有能力、有技术、有资金、有经验的务工返乡青年创办实业，带领农民致富增收。加快推进农业机械化、信息化，加强农业产前、产中、产后服务，提高农业社会化服务水平。

　　深入开展精准扶贫。以巩固拓展“双联”行动为载体，创新完善帮联、投入、协作、考核机制，集中资金、力量和项目，做到精细配置资源，精确落实政策，重点抓好100个整村推进项目建设，确保扶贫资源真正用在贫困地区、用在贫困户身上。充分发挥行业扶贫、专项扶贫、社会扶贫和各级财政“四支扶贫力量”的攻坚作用，切实用好国开行支持产业扶贫政策，扩大“双联”惠农贷款、小额担保贷款等信贷规模，努力破解资金短缺难题，力争9万人稳定脱贫。

　　（二）以两个千亿级产业链建设为牵引，持续抓好优势资源开发。围绕建设国家级重要的能源化工基地，秉持“低碳、高效、可持续”的理念，努力在石油石化、煤炭生产转化“两个千亿级产业链”延链、补链、扩链上创新创造，把庆阳最大的资源优势转化为富民兴市的经济优势。

　　推进石油石化千亿级产业链建设。按照增储上产、改造升级、合资合作、和谐共建的原则，支持油田企业高效开发，落实投资生产计划，争取原油产量达到750万吨。支持庆阳石化公司扩大生产，确保原油加工量达到350万吨以上，力争600万吨炼油升级改造项目核准并开工建设，超前谋划石油石化后续产业发展。加快“气化庆阳”等项目建设，力争年内生产天然气1.5亿立方米。强化天然气综合利用和管理，保障居民生活和交通等领域用气。

　　抓好煤炭生产转化千亿级产业链建设。支持刘园子矿井达产达标。全力推进核桃峪、新庄、马福川和甜水堡2号等4个矿井建设，争取九龙川、毛家川、钱阳山等3个矿井前期工作取得突破性进展。加快正宁电厂一期建设，确保环县电厂一期获得核准并开工建设，力争西峰热电联产项目获得核准，抓紧开展电力外送工程前期工作。持续推进晋煤集团煤、电、气一体化项目。加快煤层气实验性开采步伐，启动下游产业项目前期工作。

　　加快百万千瓦风电基地建设。确保环县毛井一期40万千瓦、甜水堡5万千瓦建成并网发电，力争毛井二期40万千瓦和山城、紫坊、乔河3个5万千瓦项目开工建设。

　　扶强做大地方工业。认真落实各项政策措施，从资金支持、财税优惠、创业基地建设、促进企业信息互联互通等方面，扶持非公经济和小微企业健康发展。积极开展千户企业扶创活动，着力解决企业融资难题，鼓励企业家和创业人士创办新企业，新增小微企业2000户以上。加快地方企业提质改造，做大做强能化集团等骨干企业，促进产品升级换代，拓宽销售渠道，增强市场竞争力。积极争取庆阳经济技术开发区获得国家批复，大力推进“一区四园”基础设施建设，主动承接央企和东部地区产业转移，重点拓展装备制造产业链，延伸食品医药产业链，打造新材料产业链，努力提高地方工业的比重。

　　（三）以开放融合为引领，持续抓好第三产业发展。抢抓国家实施新一轮对外开放的机遇，加快产业、区域、地企深度融合发展，促进第三产业发展提速、比重提高、水平提升。

　　加快文化旅游深度融合。抢抓建设华夏文明传承创新区和陇东南中医药养生保健旅游创新区的机遇，着力在规划引领、转型升级、景区建设、配套服务上下功夫。立足丰富的革命遗迹，打造红色南梁品牌；立足庆阳独有的岐黄养生文化，打造岐黄故里品牌；立足底蕴深厚的农耕文化，打造周祖圣地品牌；立足丰富多彩的民俗文化，打造民俗庆阳品牌。加快文化旅游大景区建设，完成庆阳民俗文化产业园北城墙、北门楼等主体工程，持续推进山城堡战役纪念园、太白陕甘红军纪念园、岐黄中医药文化生态园、北石窟寺、正宁调令关森林公园等景区建设。彰显地域特色，谋划开发“春季赏花、夏季避暑、秋季采摘、冬季观冰”等旅游项目。积极对接西安、延安旅游线路，努力形成辐射陇东南乃至陕甘宁的大文化、大旅游、大融合发展新格局。

　　强化区域合作交流。抢抓建设丝绸之路经济带甘肃黄金段和向西开放的战略机遇，努力打造以庆阳为重要节点、辐射带动周边地区的物流集散地。组织企业参加中西亚、中东欧国家和国内有关城市举办的特色产品展销和项目推介会，开拓丝绸之路经济带沿线国家市场，推动庆阳优质农畜产品和特色文化产品“走出去”。加强与关天、西咸经济区商贸流通、基础网络和生态建设对接，探索共同谋划产业布局、共同推进资源开发的共赢机制。

　　推动地企融合发展。按照“央企做主业、做核心，地方做辅业、做服务”的思路，重点推进能化集团煤制气、油气合作开发和装备制造、风力光伏发电等项目建设，辐射带动能源开发配套服务业快速发展，实现以产业融合促进地企融合。

　　加快发展现代服务业。持续推进市区10个专业市场和大型物流园区建设，完善农畜产品综合交易市场，努力实现商品配送中心覆盖到县，农贸市场覆盖到乡镇，农家店覆盖到村，健全城乡商贸流通网络。大力实施“电子商务进农家”工程，推动协会、品牌、物流、网店、宣传“五位一体”发展，新建100个农村电子商务示范店。加快“智慧城市”创建步伐，积极发展物联网，延伸普及宽带网。稳定住房消费，升级旅游文体消费，鼓励养老健康家政消费，促进绿色消费，培育新的消费热点。

　　扩大对外招商引资。坚持外向型战略取向，立足富集的油煤气资源、优质的农产品资源、丰厚的文化旅游资源，创新运用优势资源、优惠政策、人文环境、高效服务、良好法制环境“五个招商”模式，紧盯世界500强、国内50强企业，在引新、引高、引强上求突破，力争招商引资到位资金增长20%以上。

　　（四）以强化基础支撑为重点，持续抓好项目建设。深入实施“3341”项目工程，坚持交通、水利、电力、城镇基础建设“四措并举”，抓项目、破瓶颈，努力构建互促共进、协调发展的基础网络体系。

　　交通建设，抢抓国家重点支持中西部铁路、高速公路建设的机遇，统筹推进立体交通网络建设，确保机场航站楼、西合二级公路建成投用。开工建设银西铁路和甜罗高速、华池打扮梁至庆城高速公路，着力构建贯通南北、连接东西的交通大动脉。

　　水利建设，坚持大、中、小工程结合，“四水”齐抓，民生水利和骨干水利并重，争取葫芦河供水、正宁电厂四郎河供水工程建成投用，力争小盘河水库、葫芦河莲花寺水库、巴家嘴水库新增调蓄工程开工建设，加快马莲河枢纽工程前期工作。推进节水型社会建设，加强现有水资源综合利用，为城乡居民安全饮水和能源化工基地建设提供水资源保障。

　　电力建设，加快推进5个110千伏变电站（正宁周家、镇原金龙、西峰西庄、合水、环县）、3个330千伏变电站（环县木钵、庆城驿马、宁县长庆桥）建设，完善城乡供电网络，提升电力供应保障能力。积极探索以建设地方自备电厂为载体，以220千伏线路为主、110千伏及以下线路为辅的区域电网架构，以优惠电价吸引企业入驻，构建企地联合循环产业发展区。

　　新型城镇化建设，紧紧抓住国家推进新型城镇化的政策机遇，因地制宜，顺势而为，加快新型城镇化进程。抓好棚户区改造，强力推进市县35个片区、80万平方米的棚户区改造工程，新建保障性安居工程8933套。落实户籍制度改革政策，按照“优先解决存量、合理引导增量”的原则，建立财政转移支付、城镇建设用地增加与农业转移人口市民化挂钩机制，推进全市30多万城镇常住人口市民化，促进人口向城镇有序规模集中，力争城镇化率提高2个百分点。坚持市县乡村四轮驱动，市区突出规划引领，旧城改造抓功能提升，新区建设抓设施配套，重点抓好13条城市道路和13个城市综合体建设，加大投入，着力抓好集中供水、供热、供气管网改造等工程建设；规范居民住宅小区物业管理；加大交通整治、卫生管理和噪音污染治理力度，广泛开展市民文明素质教育提升活动，争创全国文明城市、卫生城市。县城加快旧城改造、新区开发和基础配套建设，构建一县城一特色、一风貌的格局。乡镇突出设施完善和“五化”综合治理，建设功能齐全、环境优美的小城镇。村庄突出环境治理和公共服务配套，加快美丽乡村建设，创建美丽示范村20个、环境整洁村164个，改善农村人居环境。

　　（五）以绿色发展为导向，持续抓好生态环境建设。牢固树立“既要金山银山、又要绿水青山”的理念，抢抓全省建设国家生态安全屏障综合试验区的重大机遇，积极创建山清水秀的自然生态。

　　加快生态建设。深入实施“再造一个子午岭”和“固沟保塬”两大生态工程，争取董志塬区固沟保塬综合治理工程列入国家计划立项实施，全面建成西峰雨洪集蓄保塬生态工程。加快北部荒漠化治理、百万亩土地整治和百万亩梯田工程建设，治理水土流失370平方公里，新修梯田27万亩。

　　加强环境保护。坚决不上污染项目、不做短期项目、不搞政绩项目。在重点开发区，高标准落实环评措施，严格控制高污染、高耗能项目；在限制开发区，依法加强保护；在禁止开发区，加大投入，加快修复。加强油区环境治理、城市大气污染防治、农村面源污染治理和饮用水源地保护，大力发展环保产业，扎实开展环境保护大检查，持续改善环境质量。

　　强化节能减排。以“低消耗、低排放、高效率”为目标，加强矿区标准化建设、“两废”处置和生态、水源保护，抓好重点行业、重点领域污染防治和清洁生产，提倡绿色消费，加快技术改造，淘汰落后产能，确保化学需氧量、氨氮排放量、二氧化硫排放量、氮氧化物排放量控制在省上下达的范围之内。

　　（六）以体制机制创新为核心，持续抓好重点领域改革。从群众最期盼的领域抓起，从制约发展最突出的问题改起，找准改革的主攻点和发力点，在法治轨道上推进重点领域改革。

　　加快转变政府职能，继续深化行政审批制度改革，简化行政审批事项，规范行政审批行为，做好“接、放、管”工作，加快建立行政权力清单、部门责任清单、财政专项资金管理清单和政务服务网“三张清单一张网”。深化注册资本登记制度和“先照后证”改革。分类推进事业单位改革，提升基层政务服务水平。

　　加快投融资体制机制改革，创新重点领域投融资体制机制，推动政府和社会资本合作，加强公共服务、基础设施、资源环境等薄弱环节建设，适时推出一批吸引社会资本投资的项目。改革竞争性领域财政资金投入方式，探索建立基金带动社会资本投资的模式，设立财政参股的科技创新驱动基金、产业投资引导基金、贷款融资担保基金，吸引社会资本投资科技创新驱动发展、产业结构调整优化等重点领域。

　　稳步推进混合所有制经济发展，在市场准入、政策落实、要素配置等方面，实行本地企业与招引企业、大型国企与民营企业一视同仁、同等对待，促进民企与央企、国企合作发展。支持非公经济投资参股国有企业和其它鼓励社会资本投资的项目，抓好各类中小微企业培育壮大和个体工商户转企业工作。

　　有序推动农村改革，稳步扩大农村土地承包经营权、集体经济收益分配权、宅基地用益物权“三权”综合改革试点，全面开展农村土地承包经营权确权登记工作，引导农村土地规范有序流转。大力培育和扶持种植大户、家庭农场、农业企业和农民合作社发展，让农民成为农村改革的积极参与者和真正受益者。

　　深入推进财税金融改革，全面深化预算制度改革，推进政府采购改革，完善国有资产管理，全面完成“营改增”任务。加强政府性债务管理，坚决杜绝乡村举债建设。继续实施“引行入庆”战略，加快直接融资进程，支持市内中小企业在省股权交易中心挂牌融资，支持符合条件的中小企业进入“新三板”。积极推进农村产业发展资金互助合作组织和农村产权交易平台建设，加快农村资源资产化、农村资产资本化进程。坚决打击非法集资，防范金融风险，维护金融安全。

　　推进资源配置市场化改革，争取国家、省上支持建立能源资源有偿开发制度，依据市场规则、市场价格、市场竞争配置资源，把优势资源配置给优势企业，实现效益最大化。

　　（七）以保障改善民生为根本，持续抓好社会事业发展。坚持优先保障民生投入、优先安排民生项目、优先解决民生问题，促进社会公平正义，维护社会和谐稳定。

　　统筹推进各项社会事业。促进教育提质协调发展，加强学前教育，抓好义务教育，推进高中阶段教育，深化素质教育，全面提高育人水平。深入实施“全面改薄”项目，争取改造农村薄弱学校168所。加快庆化实验学校、长庆中学、庆阳思源实验学校等重点项目建设，着力改善办学条件。加快建设庆阳职业技术学院，完善职业教育集团化发展模式。积极支持陇东学院发展，提升高等教育水平。增强科技服务能力，推进科技创新平台建设，构建以企业为主体的技术创新体系，加快培育科技型中小企业。完善创新成果转化机制，大力实施一批科技项目，让科技成果惠及人民群众。加快构建公共文化服务体系，开工建设大剧院等重点工程，加快推进传媒大厦建设，深入推动“三馆、一站、一屋”免费开放，持续开展“文化进万家”和“千台大戏送农村”活动，加强文化遗产传承保护。新建村级体育活动场所100处。提高卫生计生服务水平，全面推进公立医院改革，严格落实新农合、国家基本药物和基本公共卫生服务均等化制度，加强医德医风建设，改善就医环境。加快市妇女儿童医院、全科医师培训基地、市二院等项目建设，深入实施“城乡居民健康素养提升工程”，全力推动卫生计生事业创新发展。加强食品药品安全监管，加快乡镇食品药品监管所标准化建设，落实网格化、追溯式和风险评估监管，确保人民群众“舌尖上的安全”。更加注重产品质量安全，创建全国质量强市示范城市。抓好安全生产工作，加强道路交通、消防、校园等领域安全管理，提升应急保障和企业安全生产能力，落实企业主体责任，筑牢安全生产防线。依法做好民族宗教工作，继续实施“1+9”扶贫规划，支持民族乡村加快发展。加强国防教育、“双拥”共建和民兵预备役、人民防空工作。支持工会、共青团、妇联、科协、残联等群团组织工作。发展外事侨务、档案、地方志等事业。

　　加强就业和社会保障。认真落实就业创业和就业援助政策，大力实施产业促进就业行动，积极开发就业岗位，做好高校毕业生、失业人员、复转军人、困难家庭就业帮扶工作，确保城镇登记失业率控制在4%以内。稳定和扩大社会保障覆盖面，稳步推进机关事业单位工作人员养老保险制度改革，完善养老服务和社会救助体系，探索建立医疗救助“一站式”服务模式，全面推进社会保障“一卡通”。进一步完善全民医保体系和居民大病保险制度，深化医保付费方式改革，提升异地就医结算水平。加强企业劳动用工监管，维护劳动者合法权益。大力发展民政福利和残疾人、慈善事业，加强防灾减灾能力建设。推行新型社区管理服务模式，提升为民服务的能力。

　　扎实办好民生实事。在认真落实惠民政策、完成省上确定为民办实事的同时，全力办好9件市列民生实事：扶持1800名高校毕业生到非公企业就业；救助困难职工及农民工子女大学生1000名；为全市1360所农村中小学校和市直11所学校配备饮水设施设备；建设36个乡镇综合文化站、428个“乡村舞台”和8个县（区）文化集市；实施15个乡镇卫生院“温暖工程”；继续实行婚前医学免费检查；落实市级计划生育家庭奖励扶助政策；新建和改扩建城乡社区日间照料中心、互助老人幸福院110个；持续推进“五个百”便民工程建设。

　　全面推进法治政府建设

　　做好新常态下的政府工作，必须以党的十八届四中全会精神为指针，以创建“三个生态”为载体，以法治庆阳建设为统领，着力加强法治政府建设，努力开创政府工作新局面。

　　高扬法治精神，建设法治政府。始终把法治精神、法治思维、法治方式贯穿于政府工作的全过程，坚持在市委的领导下开展工作、在法治的轨道上推动发展。自觉接受人大依法监督、政协民主监督和行政、司法、审计、社会监督。邀请人大代表、政协委员、知名专家视察政府工作，认真听取民主党派、工商联、无党派人士和各人民团体的意见，积极办理人大代表议案和政协委员提案，健全行政机关负责人出庭应诉制度。坚持依法、科学、民主决策，健全公众参与、专家论证、风险评估、合法性审查和集体讨论决定的重大行政决策机制，全面推行政府法律顾问制度，促进决策科学化、民主化、法治化。强化“法无授权不可为、法定职责必须为”的意识，依法行政、依法办事，加强行政法规、规章和规范性文件清理工作，积极推进政府机构、权限、程序、责任法定化。理顺行政执法管理机制，着力解决权责交叉、多头执法、多层执法问题。坚持严格、规范、公正、文明执法。加大对各类违法行为的惩处力度，坚决纠正有令不行、有禁不止、失职渎职行为，坚决惩治以权谋私、以权压法、徇私枉法行为，使法律成为“高压线”、制度成为“硬约束”。深化政务公开，完善公开办事制度，公开审批流程，推进政务公开信息化建设，打造“阳光政府”。

　　创新社会治理，维护公平正义。牢固树立“以民为本、执法为民”的理念，依法保障公民人身权、财产权、基本政治权利不受侵犯，保障公民经济、文化、社会权利得到落实，着力促进社会公平正义。加大普法宣传教育力度，深入推进“六五”普法和法律“七进”活动，扩大法律援助覆盖面，教育引导广大群众做法治的忠实崇尚者、自觉遵守者和坚定捍卫者，使办事依法、遇事找法、解决问题用法、化解矛盾靠法成为广大群众的思想自觉和行动自觉。坚持系统、依法、综合、源头治理，深化平安和谐庆阳建设，健全立体化社会治安防控体系，依法打击各类违法犯罪活动，提升人民群众的安全感。健全依法维权和化解纠纷机制，大力推行“阳光信访”，加快网上信访信息系统建设，畅通群众利益诉求渠道，持续唱响改善环境“好声音”，广泛凝聚干事创业“正能量”。

　　大兴实干之风，持续改进作风。秉持“作风建设永远在路上”的理念，高标准遵守中央“八项规定”、国务院“约法三章”、省委“双十条”和市委22条实施细则等规定。巩固拓展党的群众路线教育实践活动成果，扎实开展“三严三实”专项教育。按照市委“弘扬南梁精神、推动整改落实、持续优化环境”的部署，扎实整改损害群众切身利益的突出问题，清政风、纠行风、正社风，促进作风建设常态化、法治化。建立健全一抓到底、定责问效的工作落实机制，加强对“十二五”规划目标任务落实的考核评估，以“钉钉子”的精神和奋发有为的劲头全力抓落实、干到位。坚决治庸、治散、治懒，着力解决不作为、慢作为、乱作为的问题，努力形成责任干事、为民谋事、勤奋理事、合作成事的强大合力。

　　树立清风正气，强化廉政建设。按照市委创建风清气正的政治生态的要求，教育政府工作人员讲政治、讲大局、讲规矩、讲团结，廉洁奉公，勤勉尽责。始终把廉政建设与经济工作同部署、同落实、同考核，落实“图表式分责、链条式传导、网格式覆盖、倒逼式追责”，靠实政府系统党风廉政建设主体责任，强化制度约束，层层传导压力，健全风险预警和权力运行监控机制，加强对权力集中、资金密集的部门和岗位的监管。坚持无禁区、全覆盖、零容忍，严肃查处腐败案件，筑牢法治“防线”，遏制权力“越线”，健全不敢腐、不能腐、不想腐的约束机制，努力营造简单共事、真诚相待、作风民主、自律严格的良好氛围。

　　各位代表，人民赋予重托，实干创造未来。我们一定在省委、省政府和市委的坚强领导下，在市人大、市政协的监督支持下，紧紧依靠和团结带领全市人民，开拓创新，扎实工作，圆满完成今年各项目标任务，为全面建成小康社会、建设幸福美好新庆阳作出更大贡献！
